# Comparative Effects of Photobiomodulation and Platelet‐Rich Plasma on Joint Repair in Rats With Knee Osteoarthritis

**DOI:** 10.1002/lsm.70180

**Published:** 2026-08-24

**Authors:** Anne Missrie, Gabriela Saab Sampaio, Vitória Gonzaga Acerbi, Fernando Anselmo, Ítalo Ribeiro Lemes, Ana Laura Martins de Andrade

**Affiliations:** ^1^ Faculdade Israelita de Ciências da Saúde Albert Einstein Hospital Israelita Albert Einstein São Paulo São Paulo Brazil; ^2^ Hospital Israelita Albert Einstein São Paulo São Paulo Brazil; ^3^ São Paulo State University São Paulo São Paulo Brazil; ^4^ Postgraduate Program in Biomedical Engineering Universidade Brasil São Paulo São Paulo Brazil

**Keywords:** osteoarthritis and knee, photobiomodulation, plasma rich in platelets

## Abstract

**Objective:**

This study aimed to evaluate whether the combination of photobiomodulation and platelet‐rich plasma therapy provides superior anti‐inflammatory and joint‐protective effects compared to individual treatments in an experimental model of osteoarthritis.

**Methods:**

Eighteen male Wistar rats were divided into three groups: Control, Photobiomodulation, and Photobiomodulation combined with Platelet‐Rich Plasma. Osteoarthritis was induced through two intra‐articular injections of methylated bovine serum albumin, administered 7 days apart. Treatments began 3 days after the second injection. Photobiomodulation was applied three times per week using an infrared laser (808 nm, 100 mW, 4 J total energy at two knee joint points). The combined therapy group received a single injection of platelet‐rich plasma in addition to photobiomodulation. The experiment lasted 21 days after osteoarthritis induction. Animals were then euthanized, and knee joint tissues were collected for histological analysis. Joint diameter was measured before induction, 3 days after induction, and at the end of the study.

**Results:**

All groups showed a reduction in joint swelling by the end of the experiment; however, the Control group maintained significantly larger joint diameters, indicating greater inflammation. Histological evaluations confirmed these findings: the Control group showed severe cartilage degeneration, the Photobiomodulation group had moderate damage, and the combined treatment group displayed the best preservation of cartilage structure, resembling healthy tissue.

**Conclusion:**

Isolated photobiomodulation effectively reduced in rats joint edema and inflammation progression, while its combination with Platelet‐Rich Plasma showed enhanced cartilage preservation in an experimental osteoarthritis model.

## 1 Introduction

Osteoarthritis (OA) is a joint disorder characterized by the progressive degeneration of articular cartilage and the underlying subchondral bone. Its onset is primarily associated with risk factors such as aging, overweight and obesity, direct cartilage injuries, and genetic predisposition [1]. These structural changes lead to clinical manifestations including joint pain, stiffness, reduced range of motion, and consequently, impaired mobility. Such symptoms contribute to muscle weakness and diminished physical function, resulting not only in discomfort but also in significant personal limitations that directly impact patients’ quality of life and overall functional capacity [2].

Current, clinical guidelines recommend non‐pharmacological (e.g., aquatic exercise) and pharmacological (e.g., nonsteroidal anti‐inflammatory drugs (NSAIDs) as first‐line approaches for the management of OA. In general, these therapies focus on alleviating symptoms, controlling inflammation, and slowing disease progression, with earlier treatment leading to better prognosis [1, 3].

Among the non‐pharmacological interventions, photobiomodulation (PBM) has gained attention. This intervention involves the application of low‐power, monochromatic, and coherent light that, when absorbed by tissues, can trigger a range of biological processes [4]. There is evidence that PBM may have the potential to slow OA progression, particularly by alleviating symptoms, controlling inflammation [5, 6], and modulating the expression of inflammatory mediators such as tumor necrosis factor alpha (TNF‐α) and interleukin 6 (IL‐6), as well as various metabolic substances including mucopolysaccharides (e.g., chondroitin sulfate), proteoglycans, and improved organization of chondrocytes and collagen fibers [7].

In addition, previous studies have demonstrated positive effects of PBM on cartilage tissue, including improved tissue organization and lower scores on the Osteoarthritis Research Society International (OARSI) grading system compared to control groups (CGs), indicating a less severe level of cartilage degradation. Studies have also shown that PBM promotes greater inhibition of cartilage degradation by stimulating the extracellular matrix of joint tissue, slowing the progression of the degenerative process in articular cartilage, and enhancing chondroprotective factors [8, 9].

As an additional treatment option for symptom control and slowing the progression of OA, another area that has shown significant growth is the use of intra‐articular injectable agents. Among the substances used are hyaluronic acid, autologous mesenchymal stem cells, and platelet‐rich plasma (PRP) [10, 11]. Recent studies with PRP have shown promising results by reducing inflammation and pain in experimental animal models. PRP can act on various levels, such as preventing cartilage structural deterioration, reducing inflammatory events in the synovium, and modulating serum levels of PDGF‐A and VEGF, as well as synovial tissue immunoexpression [12, 13]. However, the effectiveness of PRP remains controversial, despite promising results reported in some clinical trials.

A systematic review and meta‐analysis [14] of five randomized controlled trials previously demonstrated statistically significant benefits of PRP compared to placebo in reducing knee OA symptoms. Nonetheless, these results are not consistently supported across the literature. This inconsistency may be largely attributed to the considerable methodological variability among studies, including poorly standardized or insufficiently described protocols for PRP preparation, differences in injection regimens, outcome measures, and patient selection criteria. Such heterogeneity, combined with methodological limitations that increase the risk of bias, makes it difficult to draw definitive conclusions regarding the true clinical efficacy of PRP [15].

The literature also suggests that intra‐articular PRP injections may yield even more promising outcomes in the management of OA when combined with other agents, such as hyaluronic acid [16], or adjunctive techniques, including PBM [17] and structured physical exercise programs [18]. However, available studies, especially those focusing on PBM and exercise, remain limited and often fail to directly investigate the benefits and underlying mechanisms of these combined approaches.

In this context, it is relevant, both for the scientific literature and for clinical practice, to investigate the mechanisms of action of interventions such as PBM and PRP application. It is essential to discuss how these approaches, whether used individually or in combination, may provide benefits in the treatment of OA, by elucidating the biological processes triggered by their application. The aim of this study was to evaluate whether the addition of PRP to PBM therapy enhances therapeutic outcomes in an experimental model of OA. We hypothesized that the addition of PRP to PBM would result in improved control of inflammation and cartilage degeneration compared with PBM alone.

## 2 Materials and Methods

### 2.1 Experimental Animals

Eighteen male Wistar rats (*Rattus norvegicus* var. *albinus*; Rodentia, Mammalia), weighing between 250 and 300 g, were included in this exploratory pilot study. The animals were housed at the animal facility of the Instituto Israelita de Ensino e Pesquisa, Einstein Hospital Israelita, Morato Campus, São Paulo, Brazil. Before the experimental procedures, the animals underwent a 1‐week acclimatization period.

The rats were maintained under controlled environmental conditions, with temperature between 20°C and 26°C, controlled humidity, and a 12 h light/dark cycle, with free access to food and water *ad libitum*, according to the institutional animal care protocol. Animals were monitored daily throughout the experimental period for clinical signs, body weight changes, pain, distress, and overall well‐being.

Predefined exclusion criteria included death during the protocol, signs of infection, severe distress, failure of OA induction, or technical problems during tissue processing. No animals met these criteria and each animal was considered an experimental unit.

All surgical procedures were performed under isoflurane anesthesia to minimize pain and distress. All experimental procedures complied with international ethical guidelines for animal experimentation (NATIONAL RESEARCH CUNCIL, 1996) and were approved by the Institutional Animal Care and Use Committee (IACUC) of Einstein Hospital Israelita under protocol number 5865/2023.

### 2.2 Sample Size

Sample size was based on the study by Fan et al. [19], which used a similar OA induction model and PBM protocol. According to the authors’ power analysis based on OARSI scores, 3–4 animals per group would be sufficient to detect significant differences with 80% statistical power and an α level of 0.05. To account for biological variability and potential sample loss, six animals were allocated to each experimental group. Animals were randomly assigned by simple draw to three experimental groups (*n* = 6 per group):

- CG: animals received sham PBM, with the laser device positioned identically to the treated groups but switched off, in addition to intra‐articular saline injection in the left knee.
- PBM Group (LG): animals received active PBM associated with intra‐articular saline injection in the left knee.
- PBM + PRP Group (LPG): animals received active PBM followed by intra‐articular PRP injection in the left knee.

All animals in the CG were handled and restrained under the same experimental conditions as those in the LG and LPG groups to ensure equivalent manipulation and environmental exposure among groups. Outcome assessors, histological evaluators, and the researcher responsible for morphometric analysis were blinded to group allocation.

### 2.3 Experimental Design

The primary outcomes of this study were cartilage histopathology, assessed by qualitative morphological analysis and the OARSI score, as indicators of joint structural preservation. Secondary outcomes included joint perimeter, cartilage thickness, and chondrocyte density, which were used to further characterize inflammatory status and tissue remodeling.

Knee joint diameter was assessed using a caliper (mm) before OA induction, 3 days after induction, and on the final day of intervention. PBM was applied three times per week for a total of nine sessions. On the 21st day after the start of interventions, euthanasia was performed by intraperitoneal overdose of anesthetics (ketamine 300 mg/kg and xylazine 39 mg/kg) (Figure 1). The left knee joint was then dissected, rinsed in normal saline, fixed in 15% formalin, and embedded in paraffin for histological analysis.

**Figure 1:**
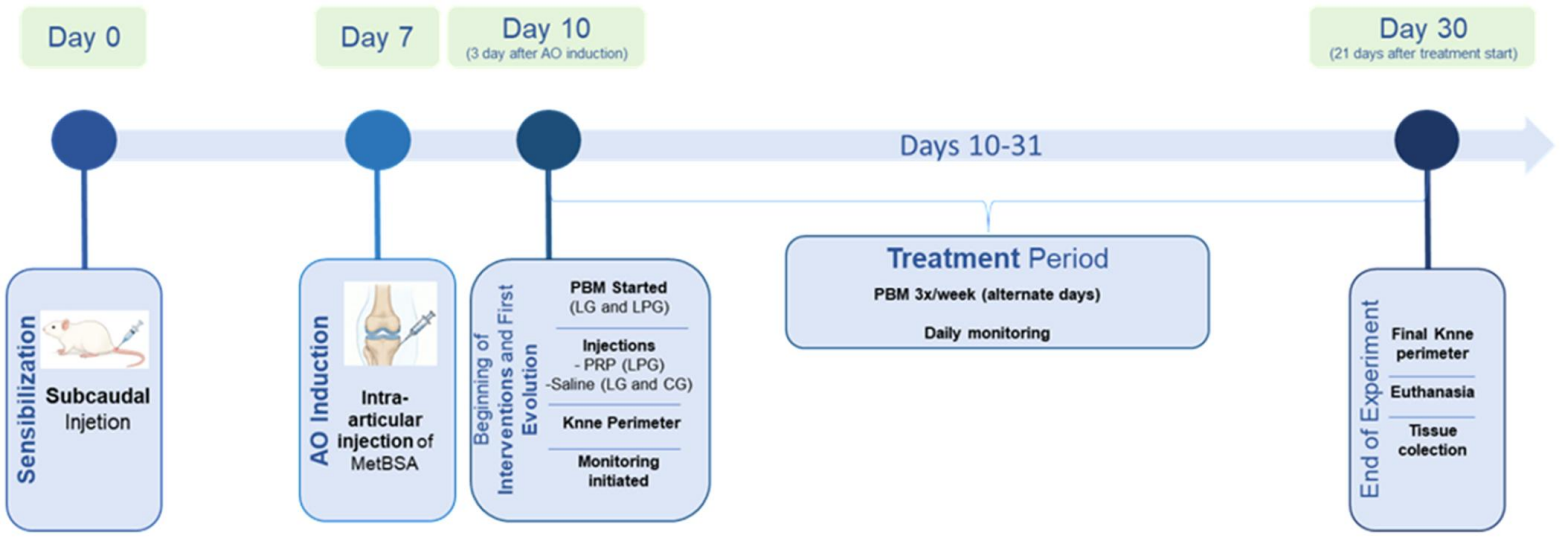
Experimental design and timeline of osteoarthritis induction, interventions, evaluations, and tissue collection in Wistar rats.

### 2.4 Induction of the OA Model

On the first day of the experiment, the animals were sensitized through a subcaudal injection containing 50 µL of methylated bovine serum albumin (MetBSA, 40 mg/mL, Sigma‐Aldrich) emulsified in 5% glucose with Complete Freund's Adjuvant (CFA), which contains *Mycobacterium sp*. (Santa Cruz Biotechnology), as described by Oliveira et al. [20]. Seven days after this initial sensitization, an intra‐articular injection was administered into the knee joint using the same solution described above, to induce an inflammatory and degradative response mimicking OA [21]. By Day 3 intra‐articular post‐injection, visible edema in the joint was observed, indicating clear signs of joint inflammation and degradation, characteristic of an osteoarthritic condition.

Prior to injections, animals were anesthetized using a combination of ketamine (7%) and xylazine (0.3%) in a 2:1 ratio, administered at a dose of 0.2 mL per 100 g of body weight. Following anesthesia, the knee joint area was shaved (trichotomy) and disinfected with povidone‐iodine (PVPI) to ensure aseptic conditions for the injection.

Animals were monitored daily for pain and distress. Analgesia was not administered to avoid potential interference with inflammatory outcomes.

### 2.5 Interventions

#### 2.5.1 PBM Application

PBM treatment started 3 days after OA induction and was performed three times per week on alternating days, for nine sessions in total. A GaAlAs (gallium‐aluminum‐arsenide) portable semiconductor laser device (DMC Equipment's, São Carlos, Brazil) was used, with an 808 nm continuous emission wavelength. Parameters were: 808 nm wavelength, 71.43 J/cm^2^ fluence per point, 2 J energy per point, and 100 mW power, as described in Table 1. The fluence value was determined by the fixed beam area of the device (0.028 cm^2^), resulting in a higher numerical value due to the small spot size of the laser. Similar parameters have been reported in previous studies using the same equipment [22]. PBM was applied to two contact points (medial and lateral) on the left knee.

**Table 1:** PBM parameters used in the study.

Parameters	
Numbers of lasers	1 *probe*
Power (mW)	100
Wavelength (nm) ± 2%	808
Power density (W/cm) ± 20%	3571.43 mW/cm^2^
Energy (J)—total	4
Energy (J)—per point	2
Energy density—per point (J/cm)	71.43 J/cm^2^
Beam diameter (cm)	0.028
Time per point (s)	20
Application mode	Perpendicular contact around the lesion

Abbreviations: cm = centimeters, J = joules, J/cm^2^ = joules per square centimeter, mW = milliwatts; nm = nanometers, s = seconds, W/cm^2^ = watts per square centimeter.

The animals were manually restrained by a trained operator to ensure accurate PBM application.

#### 2.5.2 PRP Application

For PRP application, 6 mL of blood was collected from the jugular vein using a 3 mL syringe containing acid‐citrate dextrose (ACD) solution [23]. The samples were centrifuged at 4000 rpm for 15 min at room temperature using ACD‐A tubes (BD Vacutainer, UK. Although platelet and leukocyte concentrations were not quantified, PRP preparation followed a standardized single‐spin centrifugation protocol, and the plasma fraction immediately above the buffy coat was collected to reduce leukocyte carryover. Animals in the LPG group were anesthetized with ketamine (80 mg/kg) and xylazine (10 mg/kg) and received 50 µL of PRP intra‐articularly in the left knee. Animals in the CG and LG groups were anesthetized and received 50 µL of saline intra‐articularly for control.

### 2.6 Joint Perimeter Assessment

Joint perimeter was measured using a high‐precision digital caliper (MTX Digital Caliper—150 mm) at three time points: before OA induction, 3 days after induction (to confirm the model), and at the end of the 21‐day experimental period. Measurements were taken on both knees of each animal to quantify joint edema as an indicator of local inflammation. The right knee was used as an internal control, while only the left knee, in which OA was experimentally induced and treatments were applied, was considered for the comparative analysis and presentation of results.

### 2.7 Histological Analysis

At the end of the experiment, animals were euthanized with an anesthetic overdose (ketamine 300 mg/kg and xylazine 39 mg/kg). The left knee joint was dissected, rinsed in normal saline, fixed in 15% formalin, and embedded in paraffin for histological preparation. Sections were cut at 5 μm using a micrometer system (Leica Microsystems SP 1600, Nussloch, Germany) and stained with hematoxylin‐eosin (HE) (Merck, Germany) on glass slides (Perfectalab, São Paulo, Brazil). Three predefined fields: anterior, posterior and central; were analyzed exclusively in the femoral articular cartilage for morphological and morphometric characteristics, using a microscope (Leica Microsystems AG, Wetzlar, Germany).

Samples were also histopathologically scored for OA severity using the OARSI grading system (0–6), as proposed by Pritzker et al. [24]. It is important to emphasize that scoring was performed by an experienced evaluator in a blinded manner.

### 2.8 Morphometric Analysis

HE‐stained sections were used for morphometric analysis of chondrocyte density and cartilage thickness using ImageJ 1.53t software (NIH, Bethesda, MD, USA). Analyses were performed by a blinded evaluator. Chondrocyte density was assessed exclusively in the femoral articular cartilage. For each animal, three predefined regions of interest (anterior, central, and posterior) were analyzed under ×40 magnification. Chondrocytes were manually counted using the Cell Counter plugin of ImageJ, and the mean value obtained from the three regions was considered for statistical analysis.

Cartilage thickness was measured in three regions (one central and two lateral areas) from the subchondral bone to the articular surface. The average of the three measurements obtained for each animal was used for analysis. All measurements were performed following previously published protocols [22].

### 2.9 Statistical Analysis

The data were analyzed as means and standard deviations. Normality was assessed using the Shapiro–Wilk test. Parametric variables were compared using one‐way analysis of variance (ANOVA) followed by Tukey's post hoc test. Since OARSI is an ordinal histopathological score, OARSI data were analyzed using the Kruskal–Wallis test followed by Dunn's multiple comparisons test. For the analysis of joint diameter measurements, which involved repeated measures, a two‐way ANOVA was applied to evaluate the effects of treatment, time, and their interaction. Statistical analyses were conducted using GraphPad Prism 8.0 software (GraphPad, USA). The significance level was set at 5% (*p* ≤ 0.05).

## 3 Results

The results are presented according to the predefined hierarchy of outcomes. Secondary outcomes, including joint perimeter and morphometric parameters, are presented first to characterize inflammation and tissue remodeling, followed by the primary histopathological outcomes of cartilage preservation. All animals completed the protocol and all samples were analyzed.

### 3.1 Evaluation of Joint Perimeter

Figure 2 illustrates the joint diameter of the left knee in the different groups at three time points: before induction of the experimental model, 3 days after induction, and at the end of the experimental period (21 days of intervention). It can be observed that at the time of OA model induction (Day 0), no significant differences were observed between the CG, LG, and LPG groups, with average joint diameter values around 9.3 mm.

**Figure 2:**
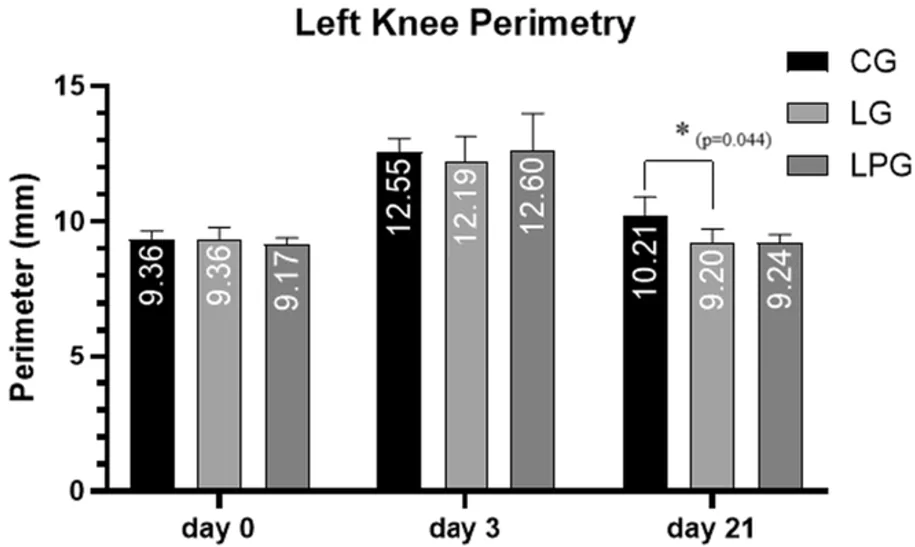
Evaluation of left knee joint perimeter before induction of the experimental model (Day 0), 3 days after model induction (Day 3), and at the end of the experimental period (final) for the CG, LG, and LPG groups. Data are presented as mean ± standard deviation. *p = 0.0449.

On Day 3, all groups showed a marked increase in joint diameter, with means exceeding 12 mm, indicating joint inflammation after induction of the experimental model.

At the end of the experimental period, a reduction in joint diameter was observed in all groups. However, CG maintained significantly higher values compared to the LG (*p* = 0.0449), suggesting greater residual inflammation. The comparison between CG and LPG showed a trend toward significance (*p* = 0.0699), while no difference was found between LG and LPG. These findings suggest that both treatments (LG and LPG) promoted a reduction in joint edema, with LG showing significantly lower joint diameter values than CG.

### 3.2 Descriptive Histological Analysis

Considering that histological characterization represented the primary outcome of the study, qualitative and semiquantitative cartilage analyses were subsequently performed.

Figure 3 presents the morphological findings obtained from the qualitative descriptive analysis of the left femoral structure in the analyzed groups. In the CG group, an intense signal of degradation was observed, with fibrillation and irregularity along the articular surface, as well as diffuse hypercellularity and disorganization of chondrocytes. In the LG group, there was a mild presence of fibrillation and surface irregularity, with moderate hypercellularity and slight chondrocyte disorganization compared to CG. In contrast, the LPG group showed a smoother surface, with few signs of fibrillation and surface irregularity. Additionally, more rounded chondrocytes and a more columnar cell organization were observed—features more consistent with a healthy articular structure.

**Figure 3:**
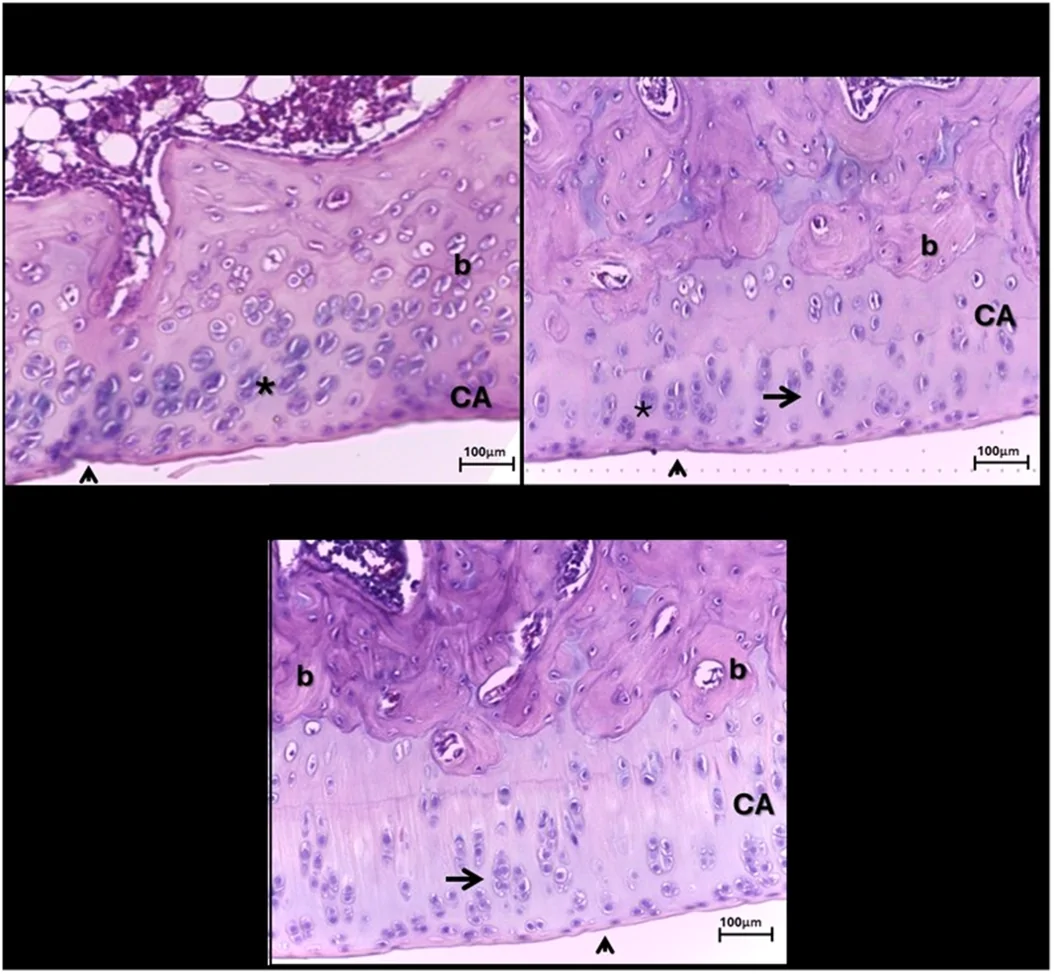
Representative photomicrographs of morphological findings from the different experimental groups (HE): CG; LG; PBM and LPG. Chondrocyte organization (**↑**); chondrocyte disorganization (asterisk); fibrillation and irregularity (arrowhead); articular cartilage (AC); subchondral bone (b) (staining: HE; scale bar: 100 μm).

In the OARSI assessment, it is possible to identify a reduction in scores in the LG and LPG groups when compared to the CG group; however, a statistically significant reduction (*p* = 0.028) was observed only between the LPG group (1.7 ± SD 0.3) and the CG group (3.3 ± SD 0.4). Although the LG group (2.2 ± SD 0.16) also showed reduced scores compared to the CG group, this difference was not statistically significant. Additionally, no significant differences were observed between the LG and LPG groups (Figure 4).

**Figure 4:**
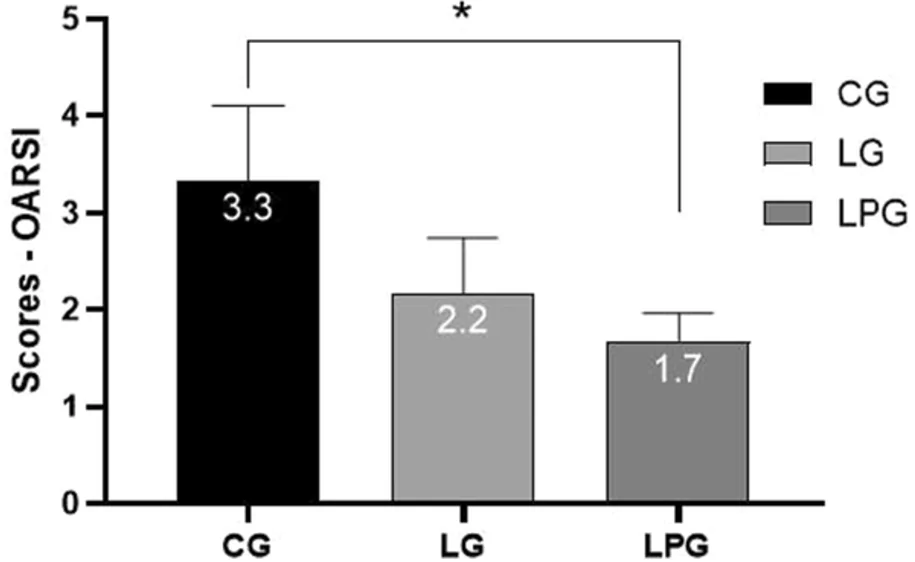
OARSI score assessment in the three experimental groups at the end of the experimental period, expressed as mean and standard deviation (*p < 0.05).

### 3.3 Morphometric Analysis

Chondrocyte counts were performed exclusively in the femur. In the analysis of chondrocyte density (Figure 5), no statistically significant differences were identified. However, a trend toward an increased number of chondrocytes can be observed in the LG (123.5 ± 20.9) and LPG (128.4 ± 15) groups compared to the CG group (101.4 ± 14).

**Figure 5:**
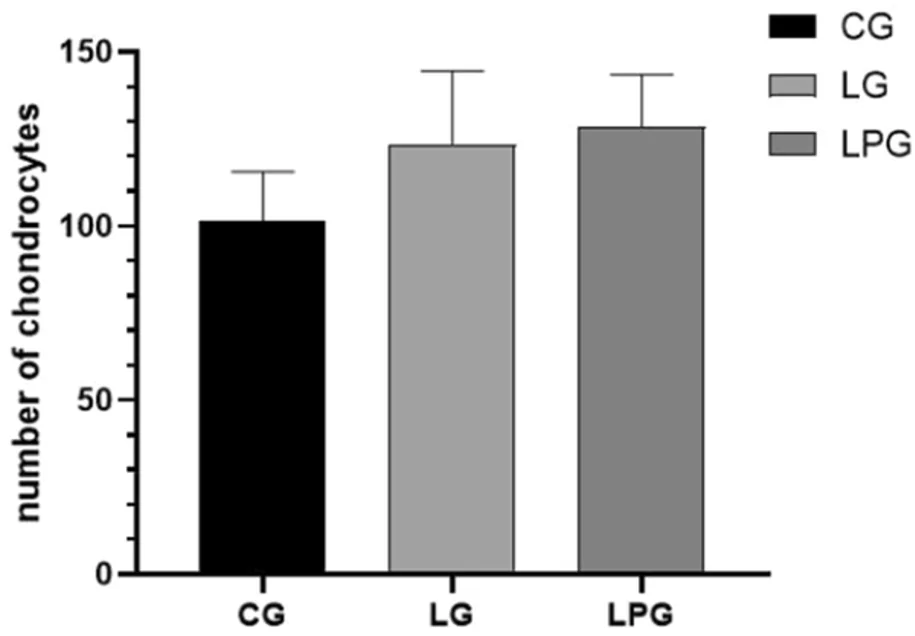
Average chondrocyte density in experimental groups after 21 days of intervention.

For the analysis of articular cartilage thickness, a significant difference (*p* = 0.0036) was observed between the CG group (34.3 ± 4.93) and the LPG group (45.3 ± 5.5). The LG group did not show statistically significant differences compared to the other groups; however, a trend toward increased mean values was observed when compared to the CG, with the LG group (41.4 ± 3.2) presenting higher values, although without reaching statistical significance (*p* = 0.064) (Figure 6).

**Figure 6:**
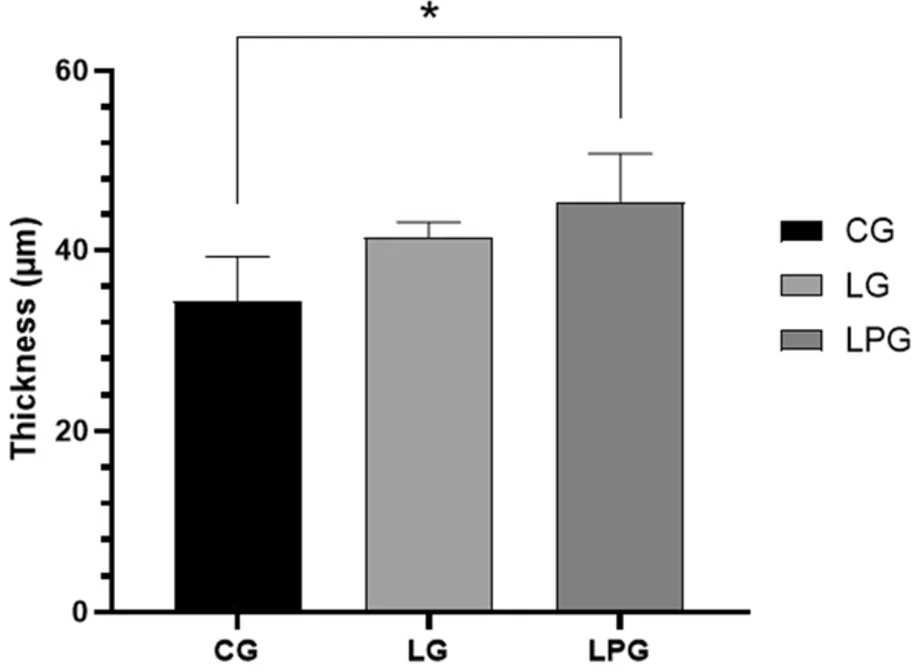
Evaluation of articular cartilage thickness at the end of the experimental period: a significant difference was observed between CG and LPG (*p = 0.0036), while the comparison between LG and CG was not significant (ns; *p* = 0.064).

## 4 Discussion

Despite numerous advances in OA treatments, both the literature and clinical practice are concise in discussing the importance and effectiveness of available non‐surgical interventions. In this context, PBM has been investigated as a promising alternative, mainly due to its anti‐inflammatory, analgesic, and tissue regeneration properties [4]. Additionally, the use of infiltration techniques, particularly local application of PRP has been the subject of considerable discussion, whether employed as a standalone intervention or in combination with other therapeutic modalities [14, 25].

The findings of this study demonstrate that application of an 808 nm PBM, using specific parameters, resulted in a significant reduction in joint diameter and an improvement in articular characteristics when compared to the CG. The literature highlights that the action of PBM in improving OA may be related to modulation of the inflammatory process and its potential to slow the progression of joint degeneration [5, 6]. Supporting the literature [26], the histological and morphometric findings suggest beneficial effects of PBM on cartilage preservation in OA. In the present study, the joint region of the PBM group (LG) appeared more preserved, with a lower degree of cartilage matrix degradation and more evident cellular organization, suggesting a protective effect of PBM on cartilage tissue compared to the CG.

These outcomes are highly relevant when considering the pathophysiology of OA, in which chondrocytes become hyperactive in an attempt to repair the ongoing damage. However, the imbalance caused by chondrocyte death activates systemic and synovial cells, such as macrophages [27], which stimulate the production of pro‐inflammatory mediators in the synovial fluid, capable of activating or inducing catabolic enzymes such as TNF‐α and reactive oxygen species (ROS) [2, 28].

Infrared PBM application activates mitochondrial cytochrome c oxidase, increasing ATP synthesis, modulating ROS and nitric oxide (NO) release, which subsequently stimulates signaling pathways related to anti‐inflammatory effects, protein synthesis, cell proliferation, and chondrogenesis, including TGF‐β mediated regulation of extracellular matrix components such as type II collagen [28]. This mechanism especially benefits chondrocytes, which are essential for maintaining the cartilage extracellular matrix. In agreement, a study applying PBM in a rat OA model observed improved cartilage recovery, with increased proteoglycans and chondrocytes in superficial layers, along with reduced calcification in deeper zones, suggesting stimulation of collagen synthesis and cellular renewal [27]. Although these mechanisms have been described in the literature, they were not directly investigated in the present study.

The selection of irradiation parameters in PBM therapy remains a topic of discussion in experimental models of OA, particularly regarding the appropriate combinations of energy and irradiation time. Previous studies have shown that PBM protocols within similar ranges can promote anti‐inflammatory effects and preservation of cartilage structure [8, 27, 28]. In the present study, the irradiation parameters were associated with morphological improvements in the articular tissue.

The therapeutic effects of PBM depend on the interaction between multiple irradiation parameters, including wavelength, power output, energy delivered, irradiation time, and beam area, rather than on a single isolated value [4]. This is particularly relevant in experimental models, where the anatomical dimensions and the characteristics of the equipment directly influence parameter selection. In point‐application systems with small spot sizes, higher power density values may be observed due to the reduced beam area, without necessarily representing excessive energy delivery. In the present study, the fixed beam area of 0.028 cm^2^ contributed to a numerically elevated power density, while the total delivered energy remained within ranges commonly reported in preclinical PBM protocols. Furthermore, the parameters used were based on previous experimental studies with the same device, supporting their applicability in this OA model [22].

Additionally, the use of PRP as a treatment for OA has been widely discussed, but few studies compare its effects when combined with PBM. Isolated PRP application has been shown in the literature to contribute to chondrocyte apoptosis inhibition, bone and vascular remodeling, and significant results in inflammation modulation and collagen synthesis [29, 30]. However, the literature is still inconclusive regarding these findings, with some studies indicating that the isolated effect of PRP may not yield significant improvements in OA, especially in more advanced stages [29].

Analyzing the results of a meta‐analysis in human studies, PRP did not show significant results in the first few months of follow‐up. However, benefits became progressively evident, reaching statistical and clinical significance by the 12th month, especially regarding function, pain, and other symptom scales. According to the study, the superiority of PRP should not be interpreted as a more intense improvement, but rather as a more long‐lasting therapeutic response. Differences compared to other intra‐articular treatments became more evident over time, as the effects of placebo or other interventions diminished [31]. It is important to emphasize that the present study was conducted in rats over a 21‐day experimental period; therefore, long‐term effects were not evaluated. Thus, although PRP may demonstrate sustained benefits over longer follow‐up periods in clinical studies, our findings are limited to the short‐term morphological effects observed in this experimental model.

In our study, both the LG and LPG groups showed a reduction in joint edema. As for morphometric analyses, it is important to note that the LPG group exhibited characteristics more similar to a healthy joint by the end of the experimental period, indicating a more favorable tissue response and better preservation of the articular structure, compared to the CG. These findings suggest that the association with PRP may have enhanced some of the effects of PBM, particularly regarding cartilage preservation.

A study conducted with medium‐sized animals showed that combined PRP and PBM treatment led to greater and longer‐lasting clinical improvements compared to isolated treatments [32]. However, in the present study, no statistically significant differences were observed between LG and LPG for several outcomes.

The PBM, when combined with other approaches such as PRP, NSAIDs, or joint supplements, has been suggested to promote longer‐lasting therapeutic effects. This combination may contribute to inflammatory modulation and tissue preservation, potentially leading to improved clinical outcomes. The therapeutic combination is potentially effective for managing OA in both humans and animals [31, 32, 33].

Thus, it is evident that both the PBM‐treated group (LG) and the PBM + PRP group (LPG) showed improvement in reducing joint edema as well as in tissue integrity observed in histological analysis, compared to the CG. These findings suggest that both treatments have the potential to modulate the inflammatory response and contribute to preserving joint structure. Furthermore, the combination of PRP with PBM appeared to promote better cartilage preservation, which may indicate a potential interaction between the two approaches.

However, it should be acknowledged that the present study was primarily focused on morphological outcomes, and no immunohistochemical or molecular analyses were performed. In addition, the relatively short experimental duration (21 days), the small sample size (*n* = 6 per group), and the lack of a group treated exclusively with PRP should be considered as limitations of this study. The platelet concentration, leukocyte content, and platelet enrichment ratio were not quantified, limiting reproducibility and comparison with previous studies. Future studies should include complete PRP characterization, a PRP‐only group, longer follow‐up periods, and molecular analyses to further elucidate the biological mechanisms underlying these therapies.

## 5 Conclusion

Isolated PBM proved effective in reducing joint edema and slowing the progression of inflammation in an experimental OA model. Moreover, the combination of PBM with PRP was associated with improved cartilage preservation, although further studies are required to clarify the underlying mechanisms.

## Ethics Statement

This study was approved by the Ethics Committee on Animal Experimentation of the Hospital Israelita Albert Einstein under Protocol No. 5865/2023.

## Conflicts of Interest

The authors declare no conflicts of interest.

## Data Availability

Data sharing not applicable to this article as no datasets were generated or analyzed during the current study.
